# SALM/Lrfn Family Synaptic Adhesion Molecules

**DOI:** 10.3389/fnmol.2018.00105

**Published:** 2018-04-05

**Authors:** Eunkyung Lie, Yan Li, Ryunhee Kim, Eunjoon Kim

**Affiliations:** ^1^Center for Synaptic Brain Dysfunctions, Institute for Basic Science (IBS), Daejeon, South Korea; ^2^Department of Biological Sciences, Korea Advanced Institute of Science and Technology (KAIST), Daejeon, South Korea

**Keywords:** adhesion molecules, synaptic, SALM, Lrfn, PSD-95

## Abstract

Synaptic adhesion-like molecules (SALMs) are a family of cell adhesion molecules involved in regulating neuronal and synapse development that have also been implicated in diverse brain dysfunctions, including autism spectrum disorders (ASDs). SALMs, also known as leucine-rich repeat (LRR) and fibronectin III domain-containing (LRFN) proteins, were originally identified as a group of novel adhesion-like molecules that contain LRRs in the extracellular region as well as a PDZ domain-binding tail that couples to PSD-95, an abundant excitatory postsynaptic scaffolding protein. While studies over the last decade have steadily explored the basic properties and synaptic and neuronal functions of SALMs, a number of recent studies have provided novel insights into molecular, structural, functional and clinical aspects of SALMs. Here we summarize these findings and discuss how SALMs act in concert with other synaptic proteins to regulate synapse development and function.

## Introduction

Synaptic adhesion molecules play important roles in the regulation of various processes involved in synapse development and function, including early axo-dendritic contacts, maturation of early synapses, synaptic transmission and plasticity, and synapse maintenance and elimination (Dalva et al., 2007; Biederer and Stagi, 2008; Han and Kim, 2008; Sanes and Yamagata, 2009; Woo et al., 2009b; Shen and Scheiffele, 2010; Siddiqui and Craig, 2011; Krueger et al., 2012; Missler et al., 2012; Valnegri et al., 2012; Takahashi and Craig, 2013; Um and Ko, 2013, 2017; Bemben et al., 2015; Ko J. et al., 2015; de Wit and Ghosh, 2016; Cao and Tabuchi, 2017; Jang et al., 2017; Krueger-Burg et al., 2017; Sudhof, 2017; Yuzaki, 2018). Prototypical examples of such molecules are neuroligins and neurexins (Sudhof, 2017). Subsequent studies have identified a large number of other synaptic molecules, suggesting that diverse synaptic adhesion molecules may act in concert to regulate synapse specificity, maturation and plasticity.

Synaptic adhesion-like molecules (SALMs), also known as leucine-rich repeat (LRR) and fibronectin III domain-containing (LRFN) proteins, are a family of synaptic adhesion molecules originally identified independently by three groups as novel cell adhesion-like molecules that bind through their C-terminal tails to the PDZ domains of PSD-95 (Ko et al., 2006; Morimura et al., 2006; Wang et al., 2006; Nam et al., 2011), an abundant excitatory postsynaptic scaffolding protein (Sheng and Kim, 2011). A total of five members of the SALM family have been identified: SALM1/Lrfn2, SALM2/Lrfn1, SALM3/Lrfn4, SALM4/Lrfn3 and SALM5/Lrfn5 (Ko et al., 2006; Morimura et al., 2006; Wang et al., 2006; Nam et al., 2011).

**Figure 1:**
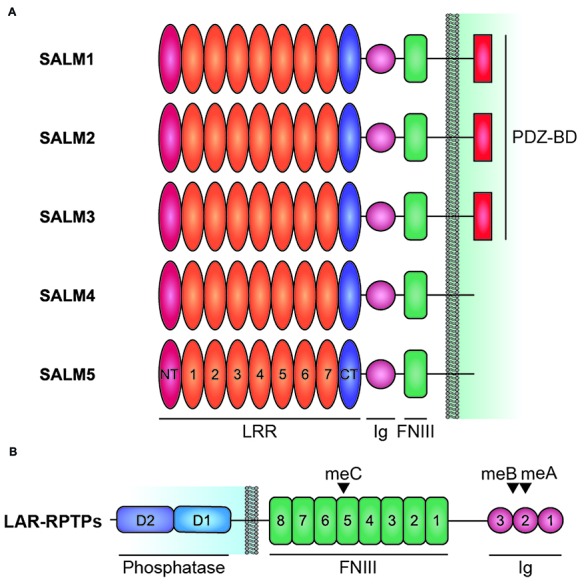
Domain structure of Synaptic adhesion-like molecules (SALMs) and LAR-RPTPs. **(A)** Domain structure of SALMs 1–5. Note that the PDZ domain-binding motif (PDZ-BD) is present in SALMs 1–3 but not in SALM4 or SALM5. FNIII, fibronectin III domain; Ig, immunoglobulin domain; LRR, leucine-rich repeats; NT and CT, N-terminal and C-terminal LRR. Note that the number of LRRs in this diagram is seven, although it was suggested to be six in early studies based on amino acid sequence analyses (Ko et al., 2006; Morimura et al., 2006; Wang et al., 2006; Nam et al., 2011). Recent X-ray crystallographic studies have identified seven LRRs in SALM5 (Lin et al., 2018) and eight LRRs in SALM2 and SALM5 (Goto-Ito et al., 2018), which may reflect different ways of defining LRRs. **(B)** Domain structure of LAR-RPTPs (LAR, PTPσ and PTPδ). D1 and D2, membrane-proximal and -distal tyrosine phosphatase domains of LAR-RPTPs; meA/B/C; mini-exon A/B/C.

These molecules share a similar domain structure, containing six LRRs, an immunoglobulin (Ig) domain, and a fibronectin type III (FNIII) domain in the extracellular side, followed by a transmembrane domain and a cytoplasmic region that ends with PDZ domain-binding motif (Figure 1A). The PDZ domain-binding motif is present in SALMs 1–3, but not SALM4 or SALM5. In contrast to the extracellular domains of SALMs, which share high amino acid sequence identities, especially in adhesion domains, the cytoplasmic regions lack shared domains and substantially differ in length as well as amino acid sequence, suggesting that they may have distinct functions.

Our previous review of SALMs summarized basic and functional characteristics of SALMs, including chromosomal locations of the corresponding genes and exon-intron structures, mRNA and protein expression patterns, protein–protein interactions, and involvement in regulating neuronal and synapse development (Nam et al., 2011). One prominent function of SALMs is to regulate neurite outgrowth and branching through mechanisms including lipid raft-associated flotillin proteins (Wang et al., 2006, 2008; Swanwick et al., 2009, 2010; Seabold et al., 2012). SALMs also regulate synapse development and function through mechanisms involving interactions with PSD-95 and glutamate receptors (Ko et al., 2006; Wang et al., 2006; Mah et al., 2010).

Notably, these functional features of SALMs have been identified mainly through *in vitro* studies. Recently, however, additional studies on SALMs using *in vivo* approaches, such as genetic mouse models, have provided intriguing insights into the physiological functions of SALMs (Li et al., 2015; Lie et al., 2016; Morimura et al., 2017). In addition, SALM3 and SALM5, which unlike other SALMs possess synaptogenic activities (Mah et al., 2010), have been found to interact trans-synaptically with presynaptic LAR family receptor tyrosine phosphatases (LAR-RPTPs; Li et al., 2015; Choi et al., 2016), a group of adhesion molecules with cytoplasmic phosphatase activity that are critically involved in various aspects of neuro- and synapse development across many species (Johnson and Van Vactor, 2003; Takahashi and Craig, 2013; Um and Ko, 2013; Figure 1B). Moreover, two independent X-ray crystallography studies have determined the stoichiometry and molecular details of the interaction of SALM5 with LAR-RPTPs (Goto-Ito et al., 2018; Lin et al., 2018). Lastly, recent clinical studies have additionally identified associations of SALMs with diverse neurodevelopmental disorders (Nho et al., 2015; Rautiainen et al., 2016; Thevenon et al., 2016; Farwell Hagman et al., 2017; Morimura et al., 2017; Bereczki et al., 2018). This review article will summarize these new findings and discuss how SALMs regulate synapse development and function.

## Synaptic Localization of SALMs

As implied by the name “synaptic adhesion-like molecule”, it was initially unclear whether SALMs are indeed localized at neuronal synapses and regulate synapse development and function through cis/trans-synaptic adhesion. The first, albeit indirect, evidence came from the fact that some SALMs directly interact with well-known excitatory synaptic proteins, such as PSD-95, N-methyl-D-aspartate receptors (NMDARs), and α-amino-3-hydroxy-5-methylisoxazole-4-propionic acid receptors (AMPARs; Ko et al., 2006; Morimura et al., 2006; Wang et al., 2006). Functionally, SALM2, artificially clustered on neuronal dendrites by antibody-coated beads, was shown to be able to recruit PSD-95 and NMDARs/AMPARs (Ko et al., 2006). In addition, SALM3 and SALM5 expressed in heterologous cells was shown to induce presynaptic differentiation in contacting axons of cocultured neurons in mixed culture assays (Mah et al., 2010), in which synaptogenic activity is tested by coculturing neurons with heterologous cells exogenously expressing synaptic adhesion molecules (Scheiffele et al., 2000; Biederer and Scheiffele, 2007).

More direct evidence for synaptic localization of SALMs has come from electron microscopy, immunocytochemistry, biochemical and proteomic analyses. One early study using immunocytochemistry detected endogenous SALM2 signals at excitatory, but not inhibitory, synapses in cultured rat hippocampal neurons (Ko et al., 2006). A subsequent electron microscopy study detected endogenous SALM4 signals at various subcellular locations in rat brain hippocampal neurons, including synaptic and extra-synaptic sites, pre- and postsynaptic sites, and dendrites and axons (Seabold et al., 2008). Biochemical experiments further demonstrated that SALMs are enriched in the postsynaptic density (PSD)—electron-dense multiprotein complexes at excitatory postsynaptic sites that contain neurotransmitter receptors, adaptor/scaffolding proteins and signaling molecules (Sheng and Sala, 2001; Sheng and Hoogenraad, 2007); SALM1 (Wang et al., 2006), SALM2 (Ko et al., 2006), SALM3 (Mah et al., 2010), SALM4 (Lie et al., 2016) and SALM5 (Mah et al., 2010).

More recently, an elegant study using proximity biotinylation, a method combining an engineered enzyme and proteomic mapping of biotinylated proteins within 10–50 nm of a particular bait protein in a subcellular environment (Han et al., 2017), identified SALMs among a large number of synaptic cleft proteins (Loh et al., 2016). Specifically, SALM1/Lrfn2 and SALM3/Lrfn4 were found to be present in the vicinity of LRRTM2 and LRRTM3, the reference excitatory synaptic adhesion molecules used in this study. Another study also using proximity biotinylation detected SALM1/Lrfn2 in close proximity to PSD-95 (Uezu et al., 2016). However, SALMs were not found to be close neighbors of the inhibitory adhesion molecules, neuroligin-2 and Slitrk3, or gephyrin (Loh et al., 2016; Uezu et al., 2016), a major inhibitory synaptic scaffolding protein (Tyagarajan and Fritschy, 2014; Choii and Ko, 2015; Krueger-Burg et al., 2017). These results suggest that some SALMs are important components of excitatory synapses; however, they do not preclude their possible presence at inhibitory synapses, since the biotinylation approach used is likely biased toward identification of more abundant proteins.

Collectively, these previous observations suggest that SALMs are present or enriched at synaptic sites, but also highlight important details that still remain to be determined, including excitatory vs. inhibitory synaptic localization of SALMs, pre- vs. postsynaptic localization, and changes in synaptic localization during development and activity. Addressing these additional questions could be aided by knockout (KO) animals combined with high-quality antibodies, as well as advanced methodologies, such as proximity biotinylation and endogenous protein tagging using CRISPR/Cas9-mediated homology-independent targeted integration (Suzuki et al., 2016).

## Trans-Synaptic Adhesions of SALMs

An early study reported that SALM3 and SALM5, but not other SALMs, expressed in heterologous cells induce presynaptic differentiation in contacting axons of cocultured neurons (Mah et al., 2010). However, it has remained unclear which presynaptic adhesion molecules mediate SALM3/5-dependent presynaptic differentiation.

**Figure 2:**
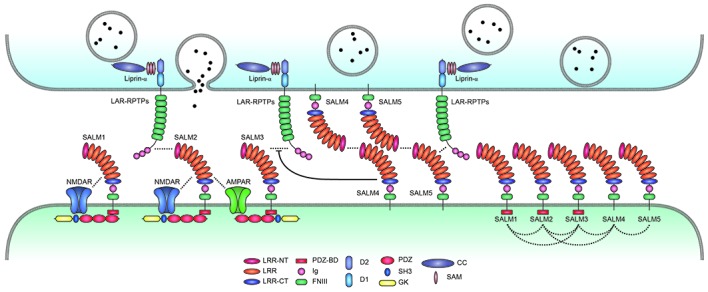
Trans-synaptic, cis-, and cytoplasmic interactions of SALMs. SALMs interact trans-synaptically with presynaptic LAR-RPTPs (LAR, PTPσ and PTPδ), in cis with AMPA/NMDA receptors and other SALM proteins, and cytoplasmically with the postsynaptic scaffolding protein PSD-95 (in the case of SALMs 1–3 but not SALM4 or SALM5). Protein interactions are indicated by the close proximity of the indicated proteins/domains or by dotted lines. Whether SALMs directly interact with NMDA/AMPA receptors remains to be determined. The trans-synaptic interactions between postsynaptic SALM3/5 and presynaptic LAR-RPTPs are known to promote presynaptic differentiation, although the function of the newly identified SALM2–LAR-RPTP (PTPδ) interaction is unclear. SALM4 interacts in cis with SALM3 to suppress the binding of SALM3 to presynaptic LAR-RPTPs and SALM3-dependent presynaptic differentiation. Postsynaptic SALM5 can also interacts with presynaptic SALM5 in a homophilic manner, which may interfere with the trans-synaptic interaction between presynaptic LAR-RPTPs and postsynaptic SALM5. The cis-interactions between different postsynaptic SALMs are based on both *in vitro* and *in vivo* results, and may be mediated by the SALM–SALM dimerization revealed by X-ray crystallographic studies. Although not shown here, some LAR-RPTPs are thought to be present and function at postsynaptic sites, in addition to presynaptic sites.

A recent study found that SALM3 interacts with presynaptic LAR-RPTPs to promote presynaptic differentiation (Li et al., 2015; Figure 2). This conclusion is supported by several lines of evidence, including protein binding, cell aggregation, and coculture assays. All three known member of the LAR-RPTP family (LAR, PTPσ and PTPδ) can interact with SALM3. Importantly, these interactions are regulated by alternative splicing of LAR-RPTPs. Specifically, the splice B insert (termed mini-exon B or meB), but not the splice A insert (meA), both of which are located in the N-terminal three Ig domains of LAR-RPTPs, is required for the interaction with SALM3 (Table 1).

**Table 1:** Influences of meA/B splice inserts in LAR-RPTPs on the interaction between LAR-RPTPs and SALMs.

Mini-exon	Interaction and change	Method	Reference
MeA	SALM3-LAR/PTPδ/PTPσ–	Purified protein binding to cells	Li et al. (2015)
	SALM5-LAR –SALM5-PTPδ/PTPσ ↓	Cell aggregation	Choi et al. (2016)
	SALM5-PTPδ –	Surface plasmon resonance	Lin et al. (2018)
	SALM5-PTPδ –	Surface plasmon resonance	Goto-Ito et al. (2018)
MeB	SALM3-LAR/PTPδ/PTPσ ↑	Protein-binding assay	Li et al. (2015)
	SALM5-LAR/PTPδ/PTPσ ↓	Cell aggregation	Choi et al. (2016)
	SALM5-PTPδ ↑	Surface plasmon resonance	Lin et al. (2018)
	SALM5-PTPδ ↑	Surface plasmon resonance	Goto-Ito et al. (2018)

*No changes, increases and decreases are indicated as horizontal bars, up arrows and down arrows, respectively*.

Like SALM3, SALM5 also interacts with LAR-RPTPs (Choi et al., 2016; Figure 2). In this case, the meB splice insert in LAR-RPTPs suppresses SALM5–LAR-RPTP interactions, an effect opposite that of meB on SALM3–LAR-RPTP interactions. Therefore, both SALM3 and SALM5 interact with LAR-RPTPs in a splicing-dependent manner, although the polarity of the modulatory effect of the insert appears to differ (but see below for conflicting results and related structural and biochemical data).

Presynaptic LAR-RPTPs are known to interact with several other postsynaptic adhesion molecules in addition to SALM3/5, including NGL-3, Slitrks, TrkC, IL1RAPL1 and IL-1RAcP (Woo et al., 2009a,b; Kwon et al., 2010; Takahashi et al., 2011, 2012; Valnegri et al., 2011; Yoshida et al., 2011, 2012; Yim et al., 2013; Li et al., 2015); also see reviews by Craig, Ko and colleagues (Takahashi and Craig, 2013; Um and Ko, 2013) for further details. These results give rise to a number of obvious questions: Why are there multiple LAR-RPTP-binding postsynaptic adhesion molecules? Does a single synapse contain all, or a majority, of the postsynaptic LAR-RPTP ligands? If so, do they compete with each other for mutually exclusive LAR-RPTP binding, or do they act in concert to fine-tune synapse regulation? These questions can also be applied to the three presynaptic LAR-RPTPs, LAR, PTPσ and PTPδ.

First, it seems unlikely that all three LAR-RPTPs are present in the same synapses, in part because LAR, PTPσ and PTPδ are differentially expressed in distinct brain regions (Kwon et al., 2010). In addition, evidence suggests that LAR, PTPσ and PTPδ differentially localize to and regulate excitatory and inhibitory synapses, with PTPσ and PTPδ being more important at excitatory and inhibitory synapses, respectively (Takahashi et al., 2011, 2012; Takahashi and Craig, 2013; Um and Ko, 2013); however, additional details remain to be determined. Splice variants of LAR-RPTPs are tightly regulated in a spatiotemporal manner (O’Grady et al., 1994; Pulido et al., 1995a,b; Zhang and Longo, 1995). In particular, each LAR-RPTP protein’s mini-exon profile, which strongly influences interactions with their postsynaptic partners (Takahashi and Craig, 2013; Um and Ko, 2013), appears to be distinct in specific brain regions. For instance, the meB splice insert in the rat hippocampus is almost always present in PTPδ, but is rarely found in LAR and is only present in about half of PTPσ molecules (Li et al., 2015), suggesting that hippocampal SALM3 is likely to interact with LAR-RPTPs in the rank order, PTPδ > PTPσ ≫ LAR (Li et al., 2015). Similarly, the majority of PTPδ splice variants in the mouse hippocampus contain the meB splice insert (Yoshida et al., 2011). Therefore, LAR-RPTPs are likely to interact with their postsynaptic partners in a spatiotemporally and molecularly regulated manner.

It can also be expected that postsynaptic LAR-RPTP ligands would be differentially expressed in specific brain regions and cell types. In addition, each postsynaptic LAR-RPTP ligand apparently has a unique preference for particular splice variants of LAR-RPTPs. For instance, meB is required for (or positively regulates) LAR-RPTP binding to SALM3, Slitrks, IL1RAPL1 and IL-1RAcP (Yoshida et al., 2011, 2012; Takahashi et al., 2012; Yim et al., 2013; Li et al., 2015), but inhibits LAR-RPTP binding to TrkC (Takahashi et al., 2011). Notably, NGL-3 differs from other postsynaptic LAR-RPTP-binding proteins in that it binds to the first two FNIII domains of LAR-RPTPs (Woo et al., 2009a), whereas all other such proteins bind to the N-terminal Ig domains of LAR-RPTPs (Takahashi et al., 2011, 2012; Yoshida et al., 2011, 2012; Yim et al., 2013; Li et al., 2015; Choi et al., 2016). This suggests the intriguing possibility that LAR-RPTPs form ternary protein complexes with NGL-3 and other postsynaptic LAR-RPTP binders, and hints at the potential interplay among these complex components. Therefore, interactions of trans-synaptic LAR-RPTPs with their postsynaptic partners likely occur in a precisely regulated manner.

It is thought that LAR-RPTPs are present mainly at presynaptic sites, because LAR proteins expressed in heterologous cells do not induce presynaptic protein clustering at contacting axons of cocultured neurons, but do induce postsynaptic protein clustering in contacting dendrites (Woo et al., 2009a). However, although some light microscopy-level immunostaining has been performed (Takahashi et al., 2011; Farhy-Tselnicker et al., 2017), clear pre- vs. postsynaptic localization of endogenous LAR-RPTPs has not been determined at the electron microscopy level. In addition, postsynaptic LAR-RPTPs have been shown to regulate dendritic spines and AMPAR-mediated synaptic transmission (Dunah et al., 2005). More recently, PTPδ coexpressed with IL1RAPL1 in cultured hippocampal neurons was found to inhibit IL1RAPL1-dependent suppression of dendritic branching, suggesting that postsynaptic PTPδ interacts in *cis* with, and inhibits, IL1RAP1 (Montani et al., 2017). Therefore, it is possible that SALM3/5-LAR-RPTP interactions also occur at postsynaptic sites in a *cis* manner.

Experiments using heterologous cells and cultured neurons have shown that SALM5 can engage in both transcellular and homophilic adhesions (Seabold et al., 2008). This suggests that presynaptic SALM5 may compete with presynaptic LAR-RPTPs for binding to postsynaptic SALM5. Alternatively, these two interactions may occur in a spatiotemporally distinct manner.

Lastly, heparan sulfate proteoglycans interact with LAR-RPTPs in the presynaptic membrane to regulate their interactions and functions (Aricescu et al., 2002; Johnson et al., 2006; Song and Kim, 2013; Coles et al., 2014; Ko J. S. et al., 2015; Farhy-Tselnicker et al., 2017; Won et al., 2017), and thus may regulate SALM–LAR-RPTP interactions and functions. In addition, LAR proteins associate with netrin-G1, a glycosylphosphatidylinositol-anchored presynaptic adhesion molecule (Nakashiba et al., 2000), at the presynaptic side when netrin-G1 is coupled with its cognate postsynaptic ligand NGL-1 (Song et al., 2013), suggesting the possibility that trans-synaptic SALM3/5–LAR-RPTP interactions is regulated by a neighboring trans-synaptic netrin-G1-NGL-1 interaction.

## Structures of SALMs in Complex With LAR-RPTPs

Although previous studies have identified interactions between SALM3/5 and LAR-RPTPs, the molecular stoichiometry and mechanistic details of these interactions have remained unclear. Two recent X-ray crystallography studies have been instrumental in resolving many of these uncertainties.

**Figure 3:**
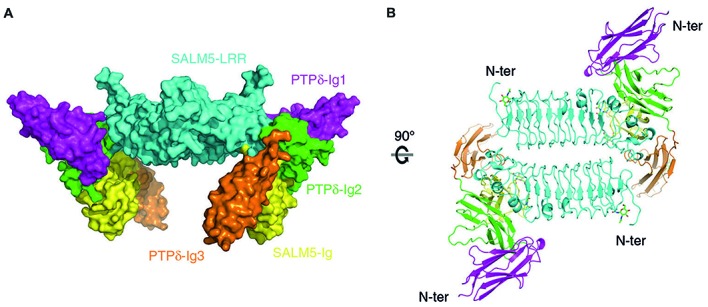
X-ray crystal structure of SALM5 in complex with PTPδ in a 2:2 heterotetrameric format. **(A)** Side view of the structure (surface representation). **(B)** Top-down view of the structure (ribbon diagram). These images were borrowed without modification from Figures 1B,C of a recent report on the crystal structure of SALM5 in complex with PTPδ (Lin et al., 2018), which are under a Creative Commons Attribution 4.0 International License (http://creativecommons.org/licenses/by/4.0/).

The first revealed that SALM5 can form a dimeric structure, in which dimerization is mediated mainly by the N-terminal LRR domain, and that this dimer forms a complex with two PTPδ monomers (Lin et al., 2018; Figures 3A,B). In this 2:2 stoichiometry, a SALM5 dimer bridges two PTPδ monomers, which are positioned at opposite sides of the SALM5 dimer. The overall shape of the complex has two components: a central platform-like structure formed by two antiparallel LRR domains of SALM5 with a concave core in its center, and four leg-like structures formed by two Ig domains of SALM5 and two Ig3 domains of PTPδ.

It was found that the specific molecular interfaces that mediate the SALM5–PTPδ interaction are the LRR domain of SALM5, which interacts with the second Ig domain of PTPδ, and the Ig domain of SALM5, which interacts with both the second and third Ig domains of PTPδ. Importantly, mutations in the LRR domain of SALM5 that disrupt dimerization were shown to abolish SALM5–LAR-RPTPs interactions and SALM5-dependent presynaptic differentiation. Therefore, SALM5 dimerization is critical for both the trans-synaptic adhesion and synaptogenic activity of SALM5.

These conclusions are further confirmed by a second study, which reported a SALM5 dimer in complex with two PTPδ monomers (Goto-Ito et al., 2018). This study identified eight LRRs whereas the other study identified seven LRRs; notably, both values differ from the number predicted in previous studies (six) based on amino acid sequence analyses (Ko et al., 2006; Morimura et al., 2006; Wang et al., 2006; Nam et al., 2011). These differences appear to reflect the specific criteria authors used to define LRRs in the different studies.

Intriguingly, this second study also solved the 2:2 structure of PTPδ in complex with SALM2 (Goto-Ito et al., 2018), a member of the SALM family that, unlike SALM3 and SALM5, has little or no synaptogenic activity (Mah et al., 2010). It is possible that SALM2 actually has synaptogenic activity that has gone unidentified in previous studies employing coculture assays and neuronal overexpression (Ko et al., 2006). Alternatively, SALM2 may interact with PTPδ to regulate other aspects of neuronal synapses. For instance, SALM2 is capable of associating with PSD-95 and NMDA/AMPARs (Ko et al., 2006). Therefore, the PTPδ–SALM2 interaction may promote postsynaptic protein clustering rather than presynaptic differentiation.

These two studies have also provided significant molecular insights into how alternative splicing regulates SALM-LAR-RPTP interactions. Specifically, they show that the meB, but not meA, splice insert is located in the junctional region between Ig2 and Ig3 domains of PTPδ, both of which are engaged in SALM5 interactions. The meB splice insert, although not directly interacting with SALM5, appears to function as a flexible linker that optimizes the position of the PTPδ-Ig3 domain for its high-affinity interaction with the SALM5-Ig domain (Goto-Ito et al., 2018; Lin et al., 2018). This conclusion is further supported by surface plasmon resonance assays that used purified PTPδ proteins, with or without meB, and demonstrated that the presence of meB increases the affinities between SALM5 and PTPδ by ~7–30 fold (Goto-Ito et al., 2018; Lin et al., 2018; Table 1).

Overall, these results are in apparent contrast with an earlier report that meB suppresses the interaction between LAR-RPTPs and SALM5 (Choi et al., 2016). A possible reason for this discrepancy is differences in the method used to assess binding—cell aggregation assays in this earlier report (Choi et al., 2016) and binding assays using purified proteins in the more recent studies (Goto-Ito et al., 2018; Lin et al., 2018). Indeed, the effects of meB on SALM3–LAR-RPTP interactions were substantially weakened in cell aggregation assays relative to protein binding assays (Li et al., 2015).

The findings of these two X-ray crystallography studies are largely similar to those investigating other LAR-RPTP interactions, which showed that meB is required for (or promotes) interactions between Slitrk1 and PTPσ (Um et al., 2014), Sltrk2 and PTPδ (Yamagata et al., 2015a), and IL1RAPL1/IL-1RAcP and PTPδ (Yamagata et al., 2015b). Therefore, these interactions, if present in the same synapse together with the SALM4–LAR-RPTP complex, are likely to be simultaneously regulated by meB.

The 2:2 stoichiometry of SALM5–LAR-RPTP interactions that involves an antiparallel LRR dimerization, something that is not observed in other LAR-RPTP-related crystal structures (Coles et al., 2014; Um et al., 2014; Yamagata et al., 2015a,b; Won et al., 2017), carries multiple potential functional implications. One possibility is that this stoichiometry could increase the affinity of the trans-synaptic SALM5–LAR-RPTP interaction. Indeed, the K_d_ values for the SALM5–PTPδ interaction determined in two independent studies ranged from 0.07 μM to 14.4 μM, indicating weaker interactions than those for LAR-RPTPs with Slitrk1, Slitrk2, IL1RAPL1, IL-1RAcP or TrkC, which are in the sub-micromolar range (0.15–0.55 μM). However, it remains unclear whether the SALM5–PTPδ interactions measured under the surface plasmon resonance condition involves the 2:2 stoichiometry.

What advantages might accrue to SALMs because they are able to achieve an appropriate trans-synaptic affinity through dimerization—a property lacking in other LAR-RPTP ligands? It is possible that a SALM5 dimer brings two PTPδ molecules close to each other to more efficiently promote presynaptic differentiation through liprin-α. Liprin-α belongs to a family of LAR-RPTP-binding scaffolding/adaptor proteins whose members are known to form homodimers and bridge LAR-RPTPs with their phospho-tyrosine protein substrates, such as β-catenin (Serra-Pagès et al., 1995, 1998; Dunah et al., 2005; Stryker and Johnson, 2007; de Curtis, 2011).

On the postsynaptic side, SALM2 dimers, which are clearly revealed in crystal structures (Goto-Ito et al., 2018), may efficiently interact with PSD-95 and PSD-95–associated proteins known to form dimeric/multimeric structures, such as Shank and Homer (Kim et al., 1996; Hsueh et al., 1997; Xiao et al., 1998; Naisbitt et al., 1999; Hayashi et al., 2009). These multimeric interactions may facilitate the formation of platform-like multi-protein structures in the PSD.

## Cis Interactions of SALMs

*Cis*-interactions of diverse synaptic adhesion molecules have received increasing attention because they often regulate trans-synaptic interactions as well as receptor-mediated synaptic transmission (Jang et al., 2017). For example, neuroligin-1 interacts in *cis* with the GluN1 subunit of NMDARs through extracellular domains to increase the synaptic abundance of NMDARs (Budreck et al., 2013). In addition, postsynaptic neurexin-1β interacts in *cis* with neuroligin-1 to suppress the trans-synaptic interaction of neuroligin-1 with presynaptic neurexins (Taniguchi et al., 2007). More recently, MDGAs (MAM domain-containing glycosylphosphatidylinositol anchors) have been found to interact in *cis* with neuroligins to modulate trans-synaptic neuroligin–neurexin interactions (Lee et al., 2013; Pettem et al., 2013; Elegheert et al., 2017; Gangwar et al., 2017; Kim et al., 2017; Thoumine and Marchot, 2017).

SALMs are involved in *cis*-interactions in addition to *trans*-interactions. The first clue came from the original study on SALMs, which reported that SALM1 associates with and promotes surface expression and clustering of NMDARs (Wang et al., 2006; Figure 2). This required the C-terminal tail of SALM1, which interacts with PSD-95 and subsequently with GluN2B subunits of NMDARs, suggesting that SALM1 indirectly interacts with and clusters NMDARs through PSD-95. However, SALM1 can also associate with GluN1, a subunit of NMDARs that lacks the cytoplasmic region, suggesting that SALM1 can directly interact with NMDARs. Additional clues for *cis*-interactions of SALMs came from the finding that bead-mediated direct clustering of SALM2 on the dendritic surface of cultured neurons induces secondary clustering of PSD-95 as well as AMPA/NMDARs (Ko et al., 2006), although whether this is mediated by direct interactions remains unclear.

A careful examination of *cis*-interactions between different SALM family members showed that all SALM members coimmunoprecipitate with each other in both a homomeric and heteromeric manner in heterologous cells (Seabold et al., 2008; Figure 2). The extracellular domains of SALMs are important for these *cis*-interactions, as evidenced by the fact that a SALM1 mutant lacking the entire cytoplasmic domain can form homo- and heteromultimers. In the brain, however, heteromeric SALM complexes are formed between SALMs 1–3, but not SALM4 or SALM5. The ability of SALM4 and SALM5, but not other SALMs, to mediate homophilic trans-synaptic adhesion suggests that postsynaptic SALMs can be segregated into three subgroups: (1) SALMs 1–3; (2) SALM4; and (3) SALM5.

However, a recent study has complicated this picture, reporting that SALM4 can coimmunoprecipitate with SALM2 in the mouse brain (Lie et al., 2016). This study further showed that SALM4 can also form complexes with SALM3 and SALM5 in heterologous cells. Additional domain-mapping experiments revealed that the LRR domain of SALM4 is important for its interactions with SALM2/5, whereas the transmembrane domain is important for its interaction with SALM3. Thus, *cis*-interactions between SALMs may be more complex than previously thought.

What might be the molecular mechanisms underlying the *cis-*interactions of SALMs? Perhaps, the aforementioned dimeric nature of SALMs revealed by X-ray crystallography may explain some of these interactions. The fact that the LRR domain of SALM4 is important for its *cis*-complex formation with SALM2/5 is consistent with the critical role of LRR domains in SALM dimerization. However, the SALM4–SALM3 *cis*-interaction, which requires the transmembrane domain, is unlikely to involve LRR dimerization.

What could be the possible functions of *cis*-interactions in SALMs? If heteromeric dimerization occurs, these interactions may increase the diversity of the subunit composition of SALM dimers. For instance, a SALM2–SALM5 dimer might bring SALM5 into proximity with the SALM2–PSD-95 complex and promote SALM5-dependent presynaptic differentiation at excitatory synapses, thereby shifting the balance of excitatory and inhibitory synapses towards excitation. In addition, these interactions may increase the diversity of non-SALM proteins, including trans-synaptic adhesion proteins, *cis*-neighboring membrane proteins, and cytoplasmic adaptor/signaling proteins around SALM complexes. This, in turn, could influence the synaptic trafficking and synapse-modulatory actions of SALMs.

## *In Vivo* Functions of SALM1/Lrfn2

As noted above, SALM1 was previously shown to be involved in surface expression and dendritic clustering of NMDARs (Wang et al., 2006). More recently, immunogold electron microscopy has revealed strong colocalization of SALM1 with the GluN1 subunit of NMDARs (Thevenon et al., 2016). These results suggest that SALM1 promotes synaptic clustering of NMDARs, although *in vivo* support for these findings has been lacking.

A recent study reported a mouse line that lacks exon 2 of the *Lrfn2* gene encoding SALM1 (*Lrfn2^–/–^* mice; Morimura et al., 2017; Table 2). Contrary to the expectation that *Lrfn2* KO would suppress synaptic NMDAR function, *Lrfn2^–/–^* mice displayed normal NMDAR-mediated synaptic transmission in the hippocampus. Instead, many SALM1-lacking synapses also lacked AMPARs, as evidenced by the slightly reduced number of dendritic spines, but markedly reduced frequency of miniature excitatory postsynaptic currents (mEPSCs), as well as altered failure rates with minimal stimulation of NMDA/AMPA-evoked postsynaptic currents (EPSCs). This suggests that many *Lrfn2^–/–^* excitatory synapses are silent synapses, an immature form of excitatory synapse that harbors NMDARs, but not AMPARs (Isaac et al., 1995; Liao et al., 1995). Therefore, *Lrfn2* KO appears to suppress synaptic delivery of AMPARs to NMDAR-only synapses during developmental synapse maturation, rather than acting at the previous step to suppress synaptic levels of NMDARs. In line with this change, *Lrfn2* KO causes an increase in NMDAR-dependent long-term potentiation (LTP), likely because silent synapses have more room to accommodate incoming AMPARs. In addition to these functional changes at excitatory synapses, *Lrfn2^–/–^* mice show morphological changes, including reduced spine head size and increased spine length (Morimura et al., 2017), suggesting that *Lrfn2* KO suppresses normal development of dendritic spines. Collectively, these findings suggest that *Lrfn2* KO suppresses both morphological and functional maturation of excitatory synapses.

**Table 2:** Main phenotypes of SALM/Lrfn-mutant mice.

Protein/gene name	Main synaptic phenotypes	Main behavioral phenotypes	Reference
SALM1/Lrfn2	Spine head size ↓	Social interaction ↓	Morimura et al. (2017)
	Spine length ↑	Repetitive behavior ↑
	Silent synapse number ↑	Acoustic startle ↑
	LTP ↑	Prepulse inhibition ↓
SALM3/Lrfn4	Excitatory synapse number ↓	Locomotor activity ↓	Li et al. (2015)
	LTP and LTD–
SALM4/Lrfn3	Excitatory and inhibitory synapse number ↑		Lie et al. (2016)
	Excitatory synapse number normalized by SALM4/SALM3 double KO

Behaviorally, *Lrfn2^–/–^* mice display autistic-like behavioral abnormalities, including suppressed social interaction and enhanced repetitive behaviors. They also show enhanced acoustic startle and suppressed prepulse inhibition, suggestive of impaired sensory-motor gating. Furthermore, using targeted gene sequencing, this study identified point mutations of the *LRFN2* gene in individuals with autism spectrum disorders (ASDs), and demonstrated that a missense mutation inhibits the association of SALM1 with PSD-95. Interestingly, *Lrfn2^–/–^* mice show enhanced spatial learning and fear memory, consistent with the enhanced LTP observed in these mice and a report that some individuals with *LRFN2* mutations show enhanced memory together with delayed speech development (Thevenon et al., 2016).

## *In Vivo* Functions of SALM3/Lrfn4

Mice carrying a null mutation of the *Lrfn4* gene (*Lrfn4^–/–^* mice) have been used to investigate *in vivo* functions of SALM3 (Li et al., 2015). These mice show reduced excitatory synapse number, as supported by spontaneous excitatory synaptic transmission and electron microscopic data, but their inhibitory synapses are minimally affected. However, NMDAR-mediated synaptic transmission and NMDAR-dependent synaptic plasticity—both LTP and LTD (long-term depression)—were unaffected in *Lrfn4^–/–^* mice.

The strong influence of *Lrfn4* KO on excitatory synapse development relative to synaptic function or plasticity is in line with the role of SALM3 as a synapse organizer that regulates presynaptic differentiation by interacting with LAR-RPTPs. SALM3/Lrfn4 was found to associate with 14-3-3 and NCK signaling adaptors to regulate actin-rich lamellipodial structures in monocytes through mechanisms involving the Rac1 small GTPase (Konakahara et al., 2011, 2012). Given that dendritic spines are actin-rich structures (Cingolani and Goda, 2008), LAR-RPTP–induced SALM3 clustering at postsynaptic sites might promote 14-3-3/NCK- and Rac1-dependent actin polymerization to promote synapse development.

Behaviorally, *Lrfn4^–/–^* mice show reduced locomotor activity in both novel and familiar environments, but exhibit normal anxiety-like behaviors. These mice also perform normally in learning and memory tests, including Morris water maze, novel object recognition, contextual fear conditioning, and T-maze spontaneous/reward alternations. The minimal effect of *Lrfn4* KO on learning and memory behaviors is in keeping with the largely normal LTP in these mice. However, it remains unclear how *Lrfn4* KO leads to behavioral hypoactivity.

SALM3 has recently been implicated in the regulation of epilepsy (Li et al., 2017). This study showed that SALM3/Lrfn4 expression is significantly increased in two distinct animal models of epilepsy, and further found that suppression of SALM3 expression by virus-mediated SALM3 knockdown ameliorates seizure activity as well as neuronal hyperexcitability. These results suggest that SALM3 promotes epileptogenesis and that its suppression has therapeutic potential.

## *In Vivo* Functions of SALM4/Lrfn3

Whether SALM4 regulates synapse development or function has remained unclear, partly because SALM4 does not have synaptogenic activity (unlike SALM3 and SALM5) or a PSD-95-binding C-terminal tail (unlike SALMs1–3). However, it should be noted that SALM4 is immunodetected in neuronal synapses in addition to dendrites and axons (Seabold et al., 2008). A recent study using mice lacking SALM4 (*Lrfn3^–/–^* mice) demonstrated that SALM4 has unexpected negative effects on the density of excitatory and inhibitory synapses (Lie et al., 2016). Specifically, *Lrfn3^–/–^* mice display increases in the number of excitatory and inhibitory synapses in the hippocampus, as supported by the density of PSDs and frequency of mE/IPSCs.

This study further addressed the mechanisms underlying the SALM4-dependent negative regulation of synapse density, reporting that postsynaptic SALM4 interacts in *cis* with SALM3, which possesses synaptogenic activity and exhibits a highly overlapping distribution pattern in the brain (Mah et al., 2010; Lie et al., 2016). This *cis*-interaction, in turn, inhibits the trans-synaptic interaction of SALM3 with presynaptic LAR-RPTPs and suppresses SALM3-dependent presynaptic differentiation (Lie et al., 2016). In support of these conclusions, coexpression of SALM4 with SALM3 in heterologous cells blocks binding of purified soluble LAR to SALM3 and inhibits SALM3-dependent presynaptic differentiation in contacting axons of cocultured neurons. Given that the transmembrane domain of SALM4 is required for *cis*-interactions with and inhibition of SALM3, it is unlikely that LRR-mediated SALM4–SALM3 heterodimerization, if it occurs, underlies the *cis*-inhibition.

Importantly, genetic support for these conclusions is provided by SALM3/SALM4 double-KO mice, in which the increased excitatory synapse density observed in SALM4 single-KO mice is normalized, as supported by both electron microscopy and mEPSC recordings (Lie et al., 2016). In contrast, double KO does not normalize the increased density of inhibitory synapses, suggesting that SALM4 negatively regulates inhibitory synapses through mechanisms independent of SALM3. Because SALM4 can also interact in *cis* with SALM5, which possesses synaptogenic activity (Mah et al., 2010), SALM5 might also play a role in SALM4-dependent regulation of inhibitory synapses. In support of this possibility, expression of SALM3 and SALM5 in heterologous cells and cultured neurons induces both excitatory and inhibitory presynaptic contacts, and SALM5 knockdown in cultured neurons suppresses both excitatory and inhibitory synapses (Mah et al., 2010). However, whereas SALM3, artificially aggregated on dendritic surfaces of cultured neurons, induces secondary clustering of PSD-95, aggregated SALM5 does not induce gephyrin clustering (Mah et al., 2010).

## SALMs in Neurodevelopmental Disorders

SALMs have been implicated in diverse neurodevelopmental and psychiatric disorders (Table 3). *LRFN2*, encoding SALM1, has recently been implicated in learning disabilities, as supported by impaired working memory and executive function in three individuals in a family with a 6p21 autosomal dominant microdeletion (~870 kb) encompassing three genes, including *LRFN2* (Thevenon et al., 2016). Similarly, levels of SALM1/LRFN2 proteins were found to be substantially decreased in postmortem brains of patients with neurodegenerative disorders associated with cognitive declines such as Alzheimer’s disease and Parkinson’s disease with dementia (Bereczki et al., 2018). In addition, single nucleotide polymorphism (SNP) analyses have linked *LRFN2* with the risk of antisocial personality disorder (Rautiainen et al., 2016). More recently, a targeted gene sequencing strategy identified missense mutations of *LRFN2* in individuals with ASD (Morimura et al., 2017), as noted above.

**Table 3:** Associations of SALMs/LRFNs with neurodevelopmental and psychiatric disorders.

Gene/protein name	Disorders	Reference
LRFN2/SALM1	Learning disability	Thevenon et al. (2016)
	Antisocial personality disorder	Rautiainen et al. (2016)
	ASD	Morimura et al. (2017)
	Schizophrenia	Morimura et al. (2017)
LRFN5/SALM5	ASD	Wang et al. (2009)
	ASD and intellectual disability	de Bruijn et al. (2010)
	ASD	Connolly et al. (2013)
	ASD	De Rubeis et al. (2014)
	ASD	Farwell Hagman et al. (2017)
	Schizophrenia	Xu et al. (2009)
	Developmental delay and seizure	Mikhail et al. (2011)
	Depression	Nho et al. (2015)

*LRFN5*, encoding SALM5, has been frequently associated with ASDs. SNP analyses have linked a chromosomal locus on 14q21.1 between *FBXO33* and *LRFN5* to a risk for ASD (Wang et al., 2009). A balanced de novo t(14;21)(q21.1;p11.2) translocation that leads to a ~10-fold reduction in the expression of *LRFN5*, located ~2 Mb from the translocation breakpoint, was identified in a 19-year-old girl with autism and intellectual disability (de Bruijn et al., 2010). In addition, a genome-wide association study showed that *LRFN5* is associated with a risk for ASDs (Connolly et al., 2013). Similar results were reported in a whole-exome sequencing study, although the association score was not high (De Rubeis et al., 2014). More recently, family-based diagnostic exome sequencing identified a point mutation (p.V572X) in *LRFN5* in an individual with ASD (Farwell Hagman et al., 2017).

*LRFN5* has also been implicated in other neurodevelopmental and psychiatric disorders. For example, an ~890-kb deletion encompassing *LRFN5* exons was identified in a girl with developmental delay, learning disability, seizures, microcephaly and receding forehead by high-resolution array comparative genomic hybridization (Mikhail et al., 2011). In addition, a high-resolution linkage analysis identified *LRFN5* among schizophrenia-related copy number variations (Xu et al., 2009). Lastly, a recent genome-wide gene- and pathway-based analysis identified *LRFN5* as one of four depression-associated genes (Nho et al., 2015). It is unclear why *LRFN5* is frequently associated with brain disorders. Although its synaptogenic activity might be a contributor, the fact that SALM3, another synaptogenic SALM, is not closely associated with brain disorders suggests against this possibility. Studies using transgenic mice lacking *Lrfn5* may provide insight into this question.

## Perspectives

Since the discovery of the SALM/Lrfn family about a decade ago, a large number of studies have elucidated basic characteristics and functional features of SALMs. Recent reports have shed additional light on the properties and functions of SALMs, identifying novel presynaptic ligands (LAR-RPTPs) of SALM3/5, resolving the crystal structures of SALMs in complex with LAR-RPTPs, elucidating the *in vivo* functions of SALMs, and revealing clinical implications of SALMs, collectively helping to better understand the functions of this protein family. However, our understanding of SALMs remains at a relatively early stage, leaving a number of questions to be explored.

For example, although SALMs can form heterodimers, and SALM dimers can explain the reported heteromeric *cis*-interactions between SALMs in the brain, it remains unclear whether SALM family members other than SALM5 and SALM2 form dimeric structures. It is also unclear whether SALMs directly interact with NMDA/AMPARs and, if so, whether these interactions regulate receptor functions or synaptic adhesions in a reciprocal manner. Because SALM3 and -5 are part of many LAR-RPTP–interacting postsynaptic adhesion molecules, whether SALM3/5 has its own unique roles, or redundant functions, remains to be determined.

*In vivo* functions of SALMs also require further exploration. It will be interesting to determine whether SALMs have distinct functions in different brain regions and cell types. Because individual SALMs have largely unique cytoplasmic regions, SALM-associated synaptic signaling pathways are likely to be quite diverse. Circuit mechanisms underlying various behavioral phenotypes of *Salm*-KO mice, in particular those associated with SALM-related developmental and psychiatric disorders, also need to be investigated. Given the rapid increase in information on the biology and pathophysiology of SALMs, the next 10 years are likely to witness a dramatic increase in our understanding of this interesting family of synaptic adhesion molecules.

## Author Contributions

EL, YL, RK and EK wrote the manuscript.

## Conflict of Interest Statement

The authors declare that the research was conducted in the absence of any commercial or financial relationships that could be construed as a potential conflict of interest.
